# On Glycerine in Flatulence, Acidity, and Pyrosis

**Published:** 1880-11

**Authors:** Sidney Ringer, William Murrell


### ON GLYCERINE IN FLATULENCE, ACIDITY, AND PYROSIS.

BY SYDNEY RINGER, M. D. AND WILLIAM MURRELL, M. D.

An old gentleman, who for many years suffered from distressing acidity, read in a daily paper that glycerine added to milk prevents its turning sour, and he reasoned thus: "If glycerine prevents milk turning sour, why should it not prevent me turning sour?" and he resolved to try the efficacy of glycerine for his acidity. The success of his experiment was complete, and whenever tormented by his old malady he cures himself by a recourse to glycerine. Indeed, he can now take articles of food from which he was previously compelled to abstain, provided always that he takes a drachm of glycerine immediately before, with, or directly after his food. He recommended this treatment to many of his friends—sufferers like himself—and one of these mentioned the above circumstances to us.

We have since largely employed glycerine, and find it not only very useful in acidity, but also in flatulence and pyrosis, and that it sometimes relieves pain. We meet with cases where flatulence, or acidity, or pyrosis is the only symptom, but more frequently these symptoms are combined. Some patients rife up huge quantities of wind without any other symptoms than depression of spirits; in others we get flatulence and acidity, one or other predominating; and we meet with others who suffer from acidity, flatulence, and also pyrosis. In all these various forms we find glycerine useful, and in the great majority of cases very useful. We do not mean to say that in all cases it is superior to other remedies for these complaints; indeed, in several instances it has only partially succeeded, where other remedies at once cured. On the other hand, in some cases glycerine speedily and completely succeeded, where the commonly used remedies for acidity and flatulence completely failed. We do not pretend to estimate its relative value to other remedies; we are only anxious to draw attention to its virtues.

Gas is in some instances formed in the stomach, in others in the large intestine, in some patients in both. Our observations were made on stomach flatulence, and as glycerine is so readily absorbed we should hardly expect that it would influence the formation of wind in the colon, except given in large doses, and when it acts as a slight laxative, and so expels the putrefying mass which forms the wind.

In some cases it removes pain and vomiting, probably, like charcoal, by preventing the formation of acrid acids, which irritate delicate and irritable stomachs.

We suggest that it acts by retarding or preventing some forms of fermentation and of putrefaction. J. Mekulics shows that glycerine prevents putrefaction of nitrogenous substances, as of blood diluted with water, which speedily decomposes at the ordinary temperature of the air. Two per cent. of glycerine retarded decomposition for twenty-four hours; 10 per cent. for five days. If the fluid were placed in the hatching oven, then 2 per cent. retarded decomposition for several hours, 10 per cent. for forty-eight hours, and 20 per cent. altogether prevented putrefaction. He also proves that glycerine destroys bacteria and prevents the formation of septic poison, though it will dissolve and preserve the septic poison itself.

Dr. E. Murk finds that 2 to 3 per cent. will delay lactic fermentation in milk from eighteen to twenty-four hours.

Burnham Wilmot, 1860, says glycerine preserves meat so that after several months' immersion the meat is sweet and can be eaten; and Demarquay proves that both animal and vegetable substances may be kept for six weeks to two months by glycerine.

Glycerine, however, does not prevent the digestive action of pepsin and hydrochloric acid; hence, whilst it prevents the formation of wind and acidity, probably by checking fermentation, it in no way hinders digestion. We administer a drachm to two drachms either before, with, or immediately after food. It may be given in water, coffee, tea, or lemon and soda water. In tea and coffee it may replace sugar, a substance which greatly favors flatulence, as, indeed, does tea in many cases. In some instances a cure does not occur till the lapse of ten days or a fortnight.

